# Structure of the Human Protein Kinase MPSK1 Reveals an Atypical Activation Loop Architecture

**DOI:** 10.1016/j.str.2007.10.026

**Published:** 2008-01-08

**Authors:** Jeyanthy Eswaran, Antonio Bernad, Jose M. Ligos, Barbara Guinea, Judit É. Debreczeni, Frank Sobott, Sirlester A. Parker, Rafael Najmanovich, Benjamin E. Turk, Stefan Knapp

**Affiliations:** 1 Structural Genomics Consortium, Botnar Research Centre, University of Oxford, Oxford OX3 7LD, United Kingdom; 2 Departamento de Inmunología y Oncología, Centro Nacional de Biotecnología, CSIC, Campus de la Universidad Autónoma de Madrid, Cantoblanco, E-28049 Madrid, Spain; 3 Department of Pharmacology, Yale University School of Medicine, New Haven, CT 06520, USA; 4 European Bioinformatics Institute, Wellcome Trust Genome Campus, Cambridge CB10 1SD, United Kingdom

**Keywords:** SIGNALING, PROTEINS

## Abstract

The activation segment of protein kinases is structurally highly conserved and central to regulation of kinase activation. Here we report an atypical activation segment architecture in human MPSK1 comprising a β sheet and a large α-helical insertion. Sequence comparisons suggested that similar activation segments exist in all members of the MPSK1 family and in MAST kinases. The consequence of this nonclassical activation segment on substrate recognition was studied using peptide library screens that revealed a preferred substrate sequence of X-X-P/V/I-ϕ-H/Y-T^∗^-N/G-X-X-X (ϕ is an aliphatic residue). In addition, we identified the GTPase DRG1 as an MPSK1 interaction partner and specific substrate. The interaction domain in DRG1 was mapped to the N terminus, leading to recruitment and phosphorylation at Thr100 within the GTPase domain. The presented data reveal an atypical kinase structural motif and suggest a role of MPSK1 regulating DRG1, a GTPase involved in regulation of cellular growth.

## Introduction

Protein phosphorylation plays a central role in regulating signaling pathways. Protein kinases are themselves therefore tightly controlled, often by modulating the conformation of the activation segment. This sequence typically contains 20–35 residues located between two largely conserved tripeptide motifs (DFG-Xn-APE). Most structural features of the activation segment are conserved among kinase structures determined to date (recently reviewed in Nolen et al., 2004). The classical activation segment of protein kinases includes the DFG motif, which forms a magnesium binding site, a short β sheet (β9), the activation loop, and the P + 1 loop. The loop regions form part of the substrate recognition site. In all known kinase structures, the activation segment is anchored by the DFG motif and β9 at the N-terminal end, as well as by the region between the P1 loop and a short helical segment (αEF) at the C-terminal end (Nolen et al., 2004). The activation segment is often unstructured in inactive protein kinases, with kinase activation generally involving conformational changes within the activation loop that result in formation of substrate binding sites. Adoption of the catalytically competent conformation is frequently triggered by phosphorylation of key residues within the activation segment. The structural analysis of this important region has therefore been a major focus for understanding the activation mechanism of protein kinases.

MPSK1 (myristoylated and palmitoylated serine/threonine kinase 1), also known as STK16 (serine/threonine kinase 16), PKL12 (protein kinase expressed in day 12 fetal liver), and Krct (kinase related to *cerevisiae* and *thaliana*), belongs to the NAK family (Numb-associated kinases). MPSK1 homologs have been described in human, mouse, *Drosophila*, nematodes, *Saccharomyces cerevisiae*, and *Arabidopsis thaliana* (Ligos et al., 1998, Stairs et al., 1998, Kurioka et al., 1998, Berson et al., 1999). The founding member of this family is the *Drosophila* kinase NAK, which plays an important role during asymmetric cell division through its interaction with Numb (Chien et al., 1998). In human, the NAK family constitutes three more members in addition to MPSK1 (AAK1 [adaptor-associated kinase], GAK [Cyclin G-associated kinase], and BIKE [BMP-2-inducible kinase]) (Manning et al., 2002). NAK family members are structurally diverse and share only weak sequence homology with other protein kinases (Chien et al., 1998).

**Figure 1:**
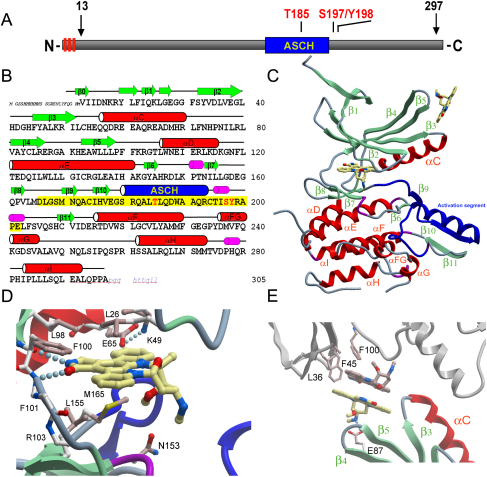
The Domain Architecture of MPSK1 (A) Schematic drawing of full-length MPSK1. Regions predicted to be membrane anchoring (N-myristoyl glycine at position 2 and S-palmitoyl cysteines at positions 6 and 8) are shown as red boxes. The location of the activation segment helix is shown in blue, and identified autophosphorylation sites are indicated. (B) Secondary structure elements labeled for MPSK1 amino acid sequence. Sheets are indicated in green, helices in red, and 3_10_ helices in magenta. The N-terminal tag sequence is indicated by small cursive letters and the C terminus not visible in the electron density is shown as small blue cursive letters. The autophosphorylation sites are highlighted in red. (C) Ribbon diagram showing an MPSK1 monomer. The two staurosporine molecules binding to the MPSK1 kinase domain are indicated using ball-and-stick representation. The activation segment is highlighted in blue. (D) Interactions of staurosporine binding to the ATP binding site. Hydrogen bonds are shown as dotted lines. The hydrogen bond between the two conserved residues Lys49 and Glu65 typically found in active kinases is also shown. (E) Detailed view of the interactions formed by the two staurosporine molecules binding between symmetry-related protein molecules.

As a consequence, MPSK1 is only distantly related to protein kinases of known three-dimensional structure, sharing only 25% sequence identity with its next structural neighbor, Aurora A. In mammals it is widely expressed, both in fetal and adult tissues, with low levels detected in skeletal muscle, heart, and spleen and high expression found in liver, testis, and kidney (Ligos et al., 1998, Stairs et al., 1998, Kurioka et al., 1998). Analysis of the 305 residue MPSK1 sequence revealed consensus sequences for N-terminal myristoylation and for palmitoylation of cysteines 6 and 8 (Berson et al., 1999) (Figure 1A). MPSK1 is localized to the Golgi apparatus but translocates to the nucleus following disorganization of the Golgi independently of MPSK1 kinase activity (Guinea et al., 2006).

MPSK1 overexpression in the mammary gland of transgenic mice results in aberrant formation of multiple terminal endbud-like structures, suggesting that the kinase might play a role in regulating stromal/epithelial interactions that occur during ductal morphogenesis (Stairs et al., 2005). In addition, MPSK1 interacts with an enzyme involved in metabolism of N-linked glycans, N-acetylglucosamine kinase (GlcNacK), and this interaction has been suggested to regulate MPSK1 kinase activity (Ligos et al., 2002). Recombinant MPSK1 is a constitutively active serine/threonine kinase capable of both autophosphorylation and phosphorylation of exogenous substrates (Ligos et al., 1998, Ligos et al., 2002, Kurioka et al., 1998, Stairs et al., 1998).

Here we report the crystal structure of human MPSK1, revealing an atypical activation segment architecture comprising a β sheet in the loop region followed by a large α-helical insert which we named activation segment C-terminal helix (ASCH). Analysis of activation segment sequences of other human kinases together with secondary structure prediction suggests that this architecture is conserved within the human NAK family and might also be present in certain members of the AGC group. In order to understand the role of this atypical structural element in substrate recognition, we determined the consensus recognition sequence of MPSK1 using degenerate peptide arrays and identified a specific interaction partner and substrate of this kinase. Our studies suggest a role for MPSK1 in regulating DRG1 (developmentally regulated GTP binding protein 1), a high molecular mass GTPase involved in the control of cellular growth and homeostasis.

## Results

### Structural Overview

Full-length MPSK1 (Figure 1A) was expressed as a soluble protein in *Escherichia coli*, but the protein oligomerized in solution and no crystals were obtained. Monodispersed protein was obtained by truncating the N terminus by 13 residues, allowing crystallization with the ATP competitive inhibitor staurosporine. The structure of the catalytic domain of MPSK1 (residues 13–297) reveals the typical bilobed domain organization of protein kinases (Figures 1B and 1C; Table 1). The asymmetric unit contains two protein molecules (A and B) which superimpose with a root-mean-square deviation (rmsd) of 1.35 Å using all main-chain atoms. However, analytical ultracentrifugation studies showed that the construct used to determine the structure is monomeric in solution (data not shown). The C termini of the two molecules differ in orientation, likely due to differences in crystal packing. Main-chain atoms of conserved structural elements of MPSK1 and its nearest structural neighbors Aurora A (Protein Data Bank [PDB] ID code: 1OL7) and PAK1 (PDB ID code: 1YHV) superimpose with an rmsd of 4.7 Å. MPSK1 also contains an additional helix between helices αF and αG (αFG; residues Asp236–Gln240). The proximity to the substrate binding site suggests that this additional structural element might play a role in substrate recognition. MPSK1 crystallized in catalytically competent conformation, as indicated by the presence of a conserved salt bridge formed by the active site Lys49 and Glu65 in helix αC and a well-ordered activation segment.

**Table 1:** Crystallographic Data and Refinement Statistics

Data Collection	
Space group	C 2 2 2_1_
Cell dimensions (Å)	104.55, 168.90, 129.73
Resolution (Å)	2.6
Unique observations (redundancy)	35,446 (7.83)
Completeness (outer shell) (%)	99.3 (94.3)
R_merge_ (%)	7.0
I/σ (outer shell)	13.52 (2.29)

Refinement	

R_work_ (R_free_) (%)	18.5 (22.93)
Protein atoms (water)	4,548 (76)
Hetero groups	4 staurosporine molecules, chloride
Rmsd bond angle (°)	1.536
Rmsd bond length (Å)	0.013
Ramachandran (%)	
Allowed	100
Generally allowed	0
Disallowed	0

### Binding of Staurosporine

MPSK1 was crystallized in the presence of the general kinase inhibitor staurosporine. Interestingly, staurosporine was found not only in the active site but also at the interface between symmetry-related molecules. The binding mode of staurosporine in the active site of MPSK1 is similar to that seen in structures of other kinases that have been described previously (Underwood et al., 2003, Bertrand et al., 2003, Schiering et al., 2003). The lactam ring of staurosporine forms two hydrogen bonds to the hinge backbone residues Pro99 and Phe101 (Figure 1D). In the crystal contact regions, two staurosporine molecules stack onto each other, forming an aromatic π interaction (Figure 1E) which has no effect on the catalytically important motifs of the protein. The first molecule binds to a hydrophobic pocket formed by Phe100 and Phe45, whereas the second staurosporine molecule forms a hydrogen bond with Glu87. This surprising finding suggests that ATP competitive inhibitors might also be used to stabilize crystal contacts and might be potentially used to also target pockets outside the kinase active site. Recently, another example of such an interaction has been reported in the crystal structure of CK1γ1 (PDB ID code: 2CMW).

### Activation Segment Structure

**Figure 2:**
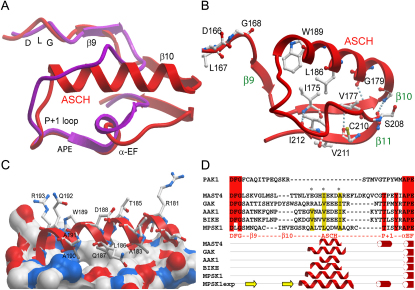
MPSK1 Activation Loop Architecture (A) Structural overlay of the activation loop of active Aurora A (PDB ID code: 1OL7) (shown in magenta) with MPSK1 (shown in red). The main structural elements are labeled. (B) Hydrogen bonds and hydrophobic interactions stabilizing the activation segment of MPSK1 are shown as dotted lines and the residues involved in stabilization are shown in ball-and-stick representation. The interacting β sheet (β11), the P + 1 loop, and the ASCH, as well as the helix αEF, are labeled. (C) Interface of the ASCH interacting with the lower kinase lobe. Hydrophobic residues are indicated as solid white surfaces. (D) Prediction of similar activation loop helices present in the kinome. Secondary structure elements predicted to be smaller than three residues have been deleted. The experimentally determined secondary structure (MPSK1exp) and the predicted one of MPSK1 are also shown. The activation segment helix and helix αEF were predicted accurately, whereas the β sheet secondary structure was not recognized by the prediction program. One representative member of the MAST kinase family predicted to contain an activation loop helix is also shown. Hydrophobic residues, buried in the interface between the ASCH and the lower kinase lobe, are indicated (^∗^) in the sequence alignment.

The activation segment architecture of MPSK1 is atypical in several ways (Figure 2A). First, the tip of the activation loop forms an extended β strand that is stabilized by antiparallel interactions with a β strand present in the C-terminal lobe of the kinase (β11). Second, the ASCH replaces the usual extended conformation at the C-terminal end of the activation segment preceding the P + 1 loop (Figure 2A). Third, rather than the conserved DFG motif, the activation segment of MPSK1 is initiated with DLG, a variant sequence found in less than 6% of human kinases. Interestingly, in the rest of the NAK family, the motif is well conserved as DFG. However, the side-chain conformation and orientation of DLG residues are similar to the ones observed in typical DFG motifs, suggesting that the MPSK1 leucine functionally replaces the conserved phenylalanine residue at this position.

Residues of the entire MPSK1 activation segment had low temperature factors and were well defined by electron density. It is likely that the central β sheet located at the tip of the activation segment locks this loop region in a rigid conformation by forming three hydrogen bonds with the antiparallel sheet inserted between the ASCH and helix αF (β11) (Figure 2B). In kinases that require phosphorylation for activity, the negative charge of the phosphate moiety compensates the high positive charge density in the activation segment region, and the catalytic loop HRD motif results in stabilization of the segment through polar interactions (Kannan and Neuwald, 2005). Interestingly, the HRD arginine (Arg147) in MPSK1 forms a large number of polar interactions with the activation segment residues Asn171 and Thr216 (side-chain oxygen) and Gln172, Asp213, and Ala173 (main-chain oxygen). Thus, in analogy to interactions typically observed in activated kinases, the interactions formed by Arg147 link the activation segment with the catalytic loop and stabilize the activation segment in a catalytically competent conformation.

In addition, a cluster of hydrophobic residues including Ile175, Val177, Val211, and Leu186 (Figure 2B) forms a small central core that also stabilizes the conformation of the activation loop. Despite this unusual architecture, the two N- and C-terminal anchor points described by Nolen et al. (2004) are conserved in the MPSK1 activation segment (Figure 2A). The ASCH is embedded in a hydrophobic cleft formed by residues of N- and C-terminal flanking regions, burying the helix residues Ala183, Leu186, and Ala191 (Figure 2C). The extensive contacts made by the MPSK1 activation segment residues suggest that it is much less dynamic than those described for other protein kinases and provides a potential rationale for why MPSK1 is constitutively active.

We were interested to explore whether MPSK1-like activation segments are present in other kinases. To this end, we examined the secondary structure predictions for all 518 human protein kinases described by Manning et al. (2002). The software used for our analysis correctly predicted the α helix present in MPSK1. Furthermore, 22 kinases were predicted to contain an α-helical region of at least four consecutive residues within the activation loop. Of the 22 predictions, 7 mapped to the ASCH region of MPSK1. The kinases predicted to share a similar helical motif include all NAK family members: AAK, BIKE, and GAK, as well as the four members of the AGC group of microtubule-associated serine/threonine kinase (MAST1–4) (Figure 2D). It is interesting to note that most hydrophobic residues making contact with the C-terminal lobe in the MPSK1 ASCH are also conserved in other NAK family members and to a certain extent also within the MAST family, lending further support to the presence of this atypical activation loop architecture among other kinases (Figure 2D).

### Substrate Specificity of MPSK1

**Figure 3:**
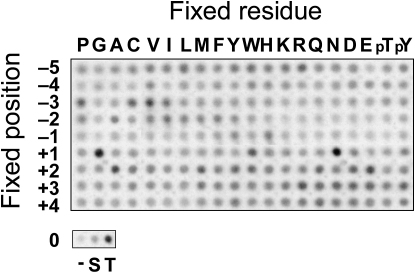
Phosphorylation Motifs for MPSK1 Biotinylated peptides bearing the shown residue at the indicated position relative to a central serine/threonine phosphoacceptor site were subjected to phosphorylation by MPSK1 using radiolabeled ATP. Aliquots of each reaction were subsequently spotted onto a streptavidin membrane, which was washed, dried, and exposed to a phosphor screen. The row marked “0” indicates either no peptide (−) or peptides with fixed Ser or Thr at the phosphoacceptor position.

The structure of the activation segment is a key determinant of substrate specificity in protein kinases. We screened a peptide library to determine the active site-mediated sequence specificity for phosphorylation by MPSK1 (Figure 3). The peptide library evaluates systematically the contribution of all amino acid residues at each of nine positions (−5 to +4) surrounding a fixed central phosphoacceptor site (Hutti et al., 2004). Reactions were performed on biotinylated peptide substrates in solution with radiolabeled ATP, followed by spotting onto a streptavidin-coated membrane. Although there was a relatively high level of background, probably due to nonspecific binding of autophosphorylated MPSK1 to the membrane, several aspects of the MPSK1 peptide phosphorylation profile were highly reproducible (Figure 3). MPSK1 appears to have a strong preference for Thr over Ser as the phosphoacceptor. There was some preference for Pro, Val, or Ile at position −3, aliphatic residues at −2, and His and Tyr at −1, as well as Asn/Gly at position +1, suggesting an optimal substrate sequence of X-X-P/V/I-ϕ-H/Y-T^∗^-N/G-X-X-X (where ϕ is an aliphatic residue).

### DRG1 Is an Interaction Partner for MPSK1

**Figure 4:**
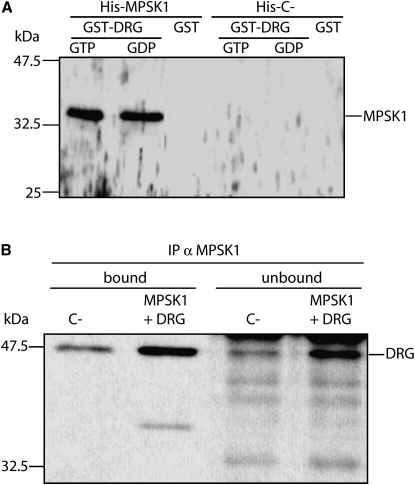
DRG1 Is an Interaction Partner for MPSK1 (A) GST pull-down experiment using recombinant His-MPSK1 and DRG1 expressed in bacteria. GST-DRG1 was saturated with the nonhydrolyzable GTP/GDP analogs, β-S-GDP or γ-S-GTP and loaded onto a glutathione-Sepharose column. After the column was stringently washed, GST-DGR1 was eluted with glutathione. Copurifying proteins were separated by SDS-PAGE and analyzed by western blotting using a specific MPSK1 antiserum. GST and a bacterial extract expressing only the His_6_ fusion partner were used as negative controls. (B) Interaction of MPSK1 and DRG1 in vivo: NIH 3T3 cells were transiently transfected with plasmids overexpressing MPSK1 and DRG1 as well as with an empty vector serving as negative control. MPSK1 was immunoprecipitated under native conditions. After extensive washing, the immunoprecipitated (bound) and the nonimmunoprecipitated fractions (unbound) were separated by SDS-PAGE and analyzed by western blot using a specific anti-DRG1 antiserum.

In order to identify potential substrates and downstream effectors of MPSK1 in vivo, we performed a yeast two-hybrid protein interaction screen. The major fraction of the positive clones (representing 77% of sequences obtained) contained sequences corresponding to the same gene, identified by sequence homology as the developmentally regulated GTP binding protein 1 (DRG1) (Sazuka et al., 1992b). The MPSK1/DRG1 interaction was also observed in GST pull-down experiments (Figure 4A); His_6_-tagged MPSK1 copurified with GST-DRG1 protein but not with GST alone. MPSK1 interacted with DRG1 independently of the presence of nonhydrolyzable β-S-GDP (GDP) or γ-S-GTP (GTP). To test whether the MPSK1/DRG1 interaction occurs in mammalian cells, NIH 3T3 cells were transiently transfected with MPSK1 and DRG1, and MPSK1 was immunoprecipitated. DRG1 was found to be significantly enriched in MPSK1 immunoprecipitates (Figure 4B), although not the entire DRG1 pool was immunoprecipitated with MPSK1. MPSK1 expressed from NIH 3T3 cells also copurified with GST-DRG1 (data not shown).

**Figure 5:**
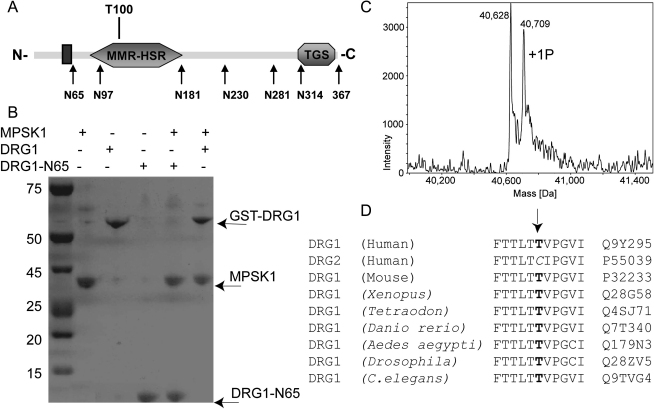
Domain Architecture of DRG1 and Phosphorylation by MPSK1 (A) DRG1 domains highlighting the glycine-rich region (black box) the GTPase domain (MMR-HSR) as well as the ThrRS, GTPase, and SpoT (TGS) domain. Boundaries of the C-terminal truncated constructs used for pull-down assays are indicated. (B) Pull-down experiment using His-MPSK1 and full-length DRG1 and DRG1-N65. Purified His-MPSK1 along with GST-DRG1 or DRG1-N65 were loaded onto the Ni-NTA column separately. After the column was stringently washed, His-MPSK1 was specifically eluted with 200 mM imidazole and the copurifying proteins were separated by SDS-PAGE. (C) ESI-MS spectrum of DRG1 treated with MPSK1, ATP, and Mg^2+^ for 1 hr at 30°C. The mass corresponding to monophosphorylated DRG1 is indicated. (D) Sequence alignment of DRG1 orthologs and human DRG2 showing the identified phosphorylation site (Thr100) (highlighted in bold). DRG2 contains a cysteine residue at that position which is highly conserved in DRG2 orthologs.

To further map the region of interaction in DRG1, we generated six GST fusion C-terminal truncation variants as shown in Figure 5A together with a schematic drawing of the DRG1 domain architecture. The interaction between MPSK1 and the truncated variants was studied in pull-down experiments using immobilized His-tagged MPSK1. All six DRG variants copurified with MPSK1, suggesting that the region 1–65 in DRG1 (DRG-N65) is sufficient for the interaction (data not shown). A pull-down experiment using full-length DRG1 and DRG-N65 is shown in Figure 5B. Unfortunately, all N-terminal deletions of DRG1 resulted only in insoluble protein, which made it impossible to rule out a contribution from regions C-terminal of DRG-N65 in interacting with MPSK1.

### DRG1 Is a Substrate for MPSK1 In Vitro

DRG1 was incubated with MPSK1 in the presence of ATP to determine whether DRG1 is a substrate of MPSK1 in vitro. We found that both the generic substrate enolase and DRG1 were rapidly phosphorylated by MPSK1 in the presence of Mg^2+^ but not Mn^2+^ (see Figure S1 in the Supplemental Data available with this article online). The strict requirement of Mg^2+^ for MPSK1-dependent phosphorylation has been noted previously (Ligos et al., 2002). Electrospray ionization mass spectrometry (ESI-MS) showed that MPSK1 rapidly phosphorylates DRG1 at one site (Figure 5C). The phosphorylation site in DRG1 was subsequently identified using a combination of proteolytic digests and mass spectrometry and was mapped to the DRG1 residue Thr100. Interestingly, Thr100 is located within the GTPase domain of DRG1, and the residue, as well as the flanking sequence, is strictly evolutionarily conserved (Figure 5D). In addition, Thr100 is not present in DRG2, which was not identified as an interaction partner of MPSK1.

### MPSK1 Autophosphorylation

**Figure 6:**
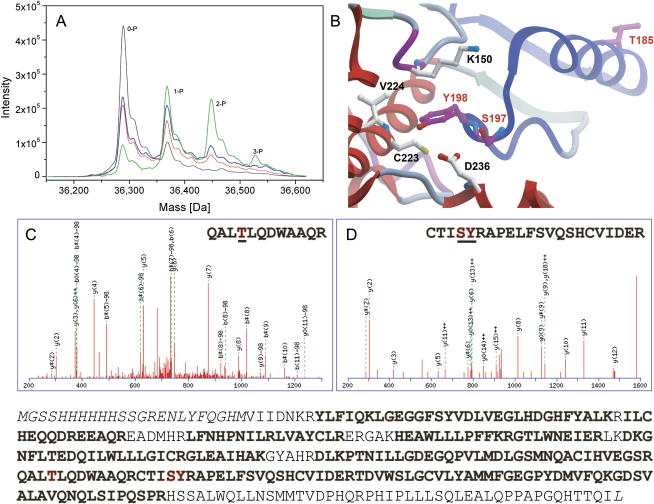
Autophosphorylation of MPSK1 and Mapping of Phosphorylation Sites (A) ESI-MS spectrum recorded for unphosphorylated MPSK1 (black line), after autophosphorylation for 1 hr (red line), 3 hr (blue), and 18 hr (green). (B–D) Location of MPSK1 autophosphorylation sites in MPSK1. Phosphorylated residues are highlighted in red and neighboring residues are shown in ball-and-stick representation. A spectrum of the two identified peptides is shown in (C) and (D), respectively. Identified peptides in MPSK1 are indicated in bold letters in the sequence shown below the panels. Phosphorylated residues are highlighted in red.

In vitro autophosphorylation of MPSK1 was followed by ESI-MS using dephosphorylated recombinant MPSK1 (Figure 6). Despite extensive treatment with λ-phosphatase and alkaline phosphatase, a small amount of monophosphorylated MPSK1 was resistant to dephosphorylation. Mass spectroscopy showed that one autophosphorylation site emerges quite rapidly (1 hr), and after 3 hr a diphosphorylated MPSK1 was clearly detected. At the end of the autophosphorylation experiment (18 hr at 30°C), MPSK1 was mainly mono- and diphosphorylated, but a third site was also evident (Figure 6A). The autophosphorylation sites were mapped by mass spectroscopy to Thr185, Tyr198, and Ser197 (Figures 6B–6D). Interestingly, Thr185 is located at the center of the ASCH and is solvent accessible (Figure 6B). However, this residue is unique to human MPSK1. Interestingly, Ser197 and Tyr198 are located in the P1 loop of MPSK1. Tyr198 is partially buried in the structure determined here, and phosphorylation at this site would require a conformational rearrangement, possibly explaining the slow phosphorylation kinetics that was observed. Tyr198 is conserved among MPSK1 homologs, and S197 is conserved only in higher eukaryotes but not in *Drosophila* or yeast.

## Discussion

Protein kinases have been classified into an evolutionary tree based on their sequence relationships (Manning et al., 2002). At a structural level, many branches of the tree remain uncharacterized. The high-resolution structure presented here is, to our knowledge, the first representing the NAK family of kinases, to which MPSK1 belongs. In keeping with its modest sequence homology with kinases of known structure, we found that MPSK1 possesses unique aspects to its structure.

The kinase domain of NAK family members, which have three homologs in *Drosophila*, two in nematodes, and four in yeast, is well conserved within this family (Figure S2). However, NAK kinases carry a number of sequence variations in otherwise conserved structural motifs of protein kinases. The consensus sequence of the catalytically important glycine-rich loop G*X*_1_G*X*_2_Φ*X*_3_ (Bossemeyer, 1994, Aimes et al., 2000) is changed to GEGGFSY in MPSK1. The presence of two adjacent glycine residues is a feature that is conserved in all NAK family members, which renders this loop region very flexible. Indeed, both glycine residues (Gly29 and Gly30) were disordered in MPSK1 (Figures S3 and S4).

Most strikingly, the structure of MPSK1 revealed an activation segment architecture containing secondary structural elements such as a β sheet at the tip of the activation segment and a large α-helical insert (ASCH) which is distinct from those found in all other known kinases (Scheeff and Bourne, 2005). However, small helical regions which usually do not exceed one helical turn are not unusual in activation segments and have been reported in inactive CDK2 (Brown et al., 1999) and Nek2 (Rellos et al., 2007), as well as in the tyrosine kinases Scr and HCK (Sicheri and Kuriyan, 1997). In addition, rather larger helical inserts are present in dimeric kinases in which activation segments have been exchanged (Oliver et al., 2007), suggesting that activation segments have some propensity to form at least transiently helical secondary structure. Prediction and sequence alignments suggested that similar structural features are likely to be present in activation segments of NAK kinase family members and might also occur in members of the MAST family of kinases.

The conformation of the activation segment is stabilized by a number of hydrophobic interactions formed with flanking structural elements as well as by an extended antiparallel β sheet formed by activation segment residues Ile175–Glu179 and a β strand located in the loop region between activation loop α helix and helix αF. It is interesting to note that smaller antiparallel β sheets are also present in active tyrosine kinases IGF1R, IRK, c-KIT, and LCK (PDB ID codes: 1K3A, 1IR3, 1PKG, and 3LCK) as well as in the serine/threonine kinases PAK1 (Favelyukis et al., 2001, Hubbard, 1997, Lei et al., 2000, Mol et al., 2003, Yamaguchi and Hendrickson, 1996). Three main-chain hydrogen bonds, typical for antiparallel β sheet structures, are conserved in activated tyrosine kinases, suggesting that this short sheet structure stabilizes the conformation of the activation segment and thus contributes to kinase activation (Figure S5). To date, the structures for only a few tyrosine kinases have been determined in their active states, so it remains to be seen whether this structural element is generally observed in active tyrosine kinases.

The kinase activation segment plays a pivotal role in both regulation and substrate recognition (Johnson et al., 1996, Nolen et al., 2004). Some kinases are activated by phosphorylation of key residues within the activation segment; in these kinases, this segment is largely disordered in the inactive and unphosphorylated state (Taylor and Radzio-Andzelm, 1994, Johnson et al., 1996). Phosphorylation stabilizes the activation segment in a conformation competent for substrate binding (Nolen et al., 2004). In the structure of unphosphorylated MPSK1, the entire activation segment is well ordered and in a conformation suitable for substrate binding, suggesting that MPSK1 is a constitutively active kinase. Biochemical assays also suggest that MPSK1 is active in the absence of additional phosphorylation. Indeed, our kinetic analysis of MPSK1 autophosphorylation showed that autophosphorylation is a slow process and that two of the three identified sites are largely buried in unphosphorylated MPSK1. However, two autophosphorylation sites are located in the P + 1 loop and phosphorylation at these locations might affect substrate recognition.

Using a peptide library, we identified sequence requirements for active site-mediated MPSK1 phosphorylation of peptide substrates. As expected based on its weak sequence homology with other serine/threonine kinases, we found that MPSK1 has an unusual phosphorylation motif that is unrelated to that of other kinases characterized thus far (Hutti et al., 2004, Pinna and Ruzzene, 1996, Songyang et al., 1994). To provide insight into the potential structural basis of MPSK1 substrate recognition, we generated a model of MPSK1 in which a consensus peptide was manually docked into the substrate site using the PKB/peptide complex as a model (Yang et al., 2002) (Figure S6). In the PKB/peptide complex, residues in substrate positions +1 to +3 bind in an extended conformation to C-terminal activation segment residues, forming three hydrogen bonds between main-chain atoms. Due to the insertion of the A loop helix, such an arrangement is not possible in MPSK1. We suggest that the backbone of MPSK1 substrates slightly bends and binds to the narrow groove between the ASCH and helix αFG. In agreement with that model, the peptide array data showed exclusion of bulky hydrophobic amino acids in position +1. Side chains in that position are oriented toward the solvent and small flexible residues like glycine or hydrophilic residues like asparagine were preferentially selected.

A striking feature of the substrate binding site is created by the MPSK1-specific helix αFG and Phe239, which together form a deep substrate binding cavity. Residues Phe239, Thr152, Tyr198, and Pro151 form a deep cleft at the bottom of that cavity. The general selection for aliphatic (at the −3 and −2 positions) and aromatic (at the −1 position) residues by MPSK1 is consistent with the overall hydrophobic nature of this substrate binding pocket. However, the identified phosphorylation site in DRG1 is not a particularly good match to the determined consensus sequence for peptides. However, substrate specificity in kinases is often determined by sites that are distant to the substrate binding site (Brown et al., 2007). In this study, we were able to show that DRG1 contains an N-terminal docking site that recruits DRG1 to MPSK1. It is likely that this interaction also determines substrate specificity leading to phosphorylation on the DRG1 residue Thr100, a phosphorylation site that is distal to the interaction domain.

The detection of autophosphorylation on tyrosine was also a surprising finding. A recent report suggested that the serine/threonine kinase DYRK (dual-specificity tyrosine phosphorylation-regulated protein kinase) has tyrosine kinase activity during folding of the protein but not as a mature protein (Lochhead et al., 2005). Because we were not able to completely dephosphorylate MPSK1, the detected tyrosine phosphorylation could similarly arise from phosphorylation during protein translation and might not be a property of mature MPSK1. The slow phosphorylation kinetics, high enzymatic activity of unphosphorylated MPSK1, and the location of the phosphorylation sites suggest that the identified autophosphorylation sites are not relevant for MPSK1 enzymatic activity.

In this study, we identified the GTP binding protein DRG1 as an interaction partner and specific substrate for MPSK1 in vivo and in vitro. DRG1 was first identified as a protein highly expressed during embryonic development in mice, but it is also present in most adult tissues (Sazuka et al., 1992a, Sazuka et al., 1992b). DRG1 is a member of the ODN family of G proteins (Park et al., 2001), which play a critical role regulating cell growth and homeostasis (Ko et al., 2004, Ishikawa et al., 2005) and are conserved from archaea to eukaryotes (Leipe et al., 2002, Caldon et al., 2001). In contrast to archaea, which harbor only a single DRG gene, eukaryotes express two closely related DRG proteins (DRG1 and DRG2). DRG1 has been shown to be downregulated in SV40-transformed cells (Schenker et al., 1994) and might cooperate with Ras and Myc in transformation of rat embryonic fibroblasts (Mahajan et al., 1996). DRG1 but not DRG2 interacts with the oncoprotein SCL/TAL1, a basic helix-loop-helix protein that is overexpressed in acute lymphoblastic leukemic cells and is involved in hematopoietic development (Mahajan et al., 1996, Zhao and Aplan, 1998). Thus, despite their high sequence similarity, DRG1 and DRG2 have different functions (Ishikawa et al., 2005), and they bind to two distinct regulatory proteins (DRFRP1 and DRFRP2, respectively) (Ishikawa et al., 2005). Only DRG1 was identified as an interaction partner of MPSK1 in yeast two-hybrid screens. In addition, the phosphorylation site identified in DRG1 is strictly conserved in eukaryotic DRG1 orthologs but is not present in DRG2.

It is noteworthy that DRG1 is stabilized in vivo by both SLC/TAL1 and DRFRP1, possibly through regulating the interaction of DRG1 with the ubiquitin system (Mahajan et al., 1996). It is therefore likely that MPSK1 regulates DRG1 function by either phosphorylation or through competition with other DRG1 binding partners. However, further experimentation will be needed to unravel the mechanisms by which MPSK1 regulates DRG1 and its role in signal transduction pathways in general.

## Experimental Procedures

### Cloning and Purification of Proteins

MPSK1 (residues 13–305) (GenBank accession number gi4505837) was subcloned into a pET-21a-derived vector (p11) in-frame with a TEV-cleavable (^∗^) N-terminal 6× His tag (MHHHHHHSSGVDLGTENLYFQ^∗^SM). Transformed *E. coli* (BL21[DE3]) were grown at 37°C in Luria-Bertani medium containing 100 μg/ml ampicillin until the OD_600_ reached 0.3 and then transferred to 18°C. Protein expression was induced at an OD_600_ of 0.8 using 1 mM isopropyl-thio-β-D-galactopyranoside for 12–16 hr. Cells were lysed in 50 mM HEPES (pH 7.5), 500 mM NaCl, 1 mM PMSF, and 0.5 mM TCEP using an EmulsiFlex high-pressure homogenizer, cleared by centrifugation at 4°C, and loaded onto a 5 ml column of nickel-Sepharose affinity resin. The column was washed with ten volumes of loading buffer (50 mM HEPES [pH 7.5], 500 mM NaCl, 5 mM imidazole, 0.5 mM TCEP, 5% glycerol) and ten volumes of wash buffer (50 mM HEPES [pH 7.5], 500 mM NaCl, 20 mM imidazole, 0.5 mM TCEP, 5% glycerol). MPSK1 was eluted by applying 5 ml portions of elution buffer containing the binding buffer with increasing concentrations of imidazole (50, 100, 250 mM). The protein was treated with λ-phosphatase for 12 hr at 4°C and diluted 10-fold into buffer (50 mM HEPES [pH 7.5]) and purified further by ion-exchange chromatography using a 1 ml HiTrapQ column. MPSK1 was eluted with a salt gradient and further purified by gel filtration using an S75 column equilibrated in 50 mM HEPES (pH 7.5), 100 mM NaCl. Purified MPSK1 was homogeneous as assessed by SDS-PAGE and ESI-MS.

DRG1 and DRG1 C-terminal truncated constructs were cloned into pGTvLI-SGC and expressed in *E. coli* (DE3). Bacterial extracts were loaded, cleared by centrifugation at 4°C, and loaded onto glutathione-Sepharose 4B (Amersham Pharmacia Biotech) affinity resin and eluted with 50 mM Tris (pH 8.0), 150 mM NaCl, and 10 mM glutathione after extensive washing using 50 mM Tris (pH 8.0), 150 mM NaCl, and 1 mM glutathione. The GST tag of DRG1-N65 was removed by treating the purified protein overnight with TEV at 4°C.

### Crystallization

Crystals were obtained by vapor diffusion at 4°C using a reservoir solution of 1.1 M ammonium sulfate, 1% PEG 3350, and 0.1 M bis-Tris (pH 5.0) by mixing 500 nl of dephosphorylated MPSK1 protein (7.1 mg/ml) containing 1 mM staurosporine and 500 nl of the reservoir solution.

### Data Collection, Structure Solution, and Refinement

Data were collected at Swiss Light Source beamline X10 to a resolution of 2.3 Å. The structure was solved with molecular replacement using Phaser (Storoni et al., 2004) with the human Aurora kinase (PDB ID code: 1OL7) as a search model. Iterative rounds of rigid-body refinement and restrained refinement with translation-liberation screw (TLS), against maximum-likelihood targets, were interspersed by manual rebuilding of the model using Coot (Emsley and Cowtan, 2004) and XtalView/Xfit (McRee, 1999). The structure was deposited in the Protein Data Bank under ID code 2BUJ.

### Yeast Two-Hybrid Screening

HF7c yeast was stably transformed with the GAL4bd-MPSK1 plasmid (pGBT8-MPSK1); an NIH 3T3 library fused to the GAL4 activator domain in the pGAD424 plasmid was then transformed and screened in minimal medium. Several clones were selected that grew under the selection conditions and also expressed *lacZ*, the second transactivable marker gene. pGAD424 plasmids harbored by the positive yeast clones were isolated, and positive interaction was confirmed by individual cotransformation of HF7c yeast with the pGBT8-MPSK1 plasmid or the negative controls pGBT8-p16 (12) and pGBT8. Positive clones were isolated and fully sequenced.

### Rabbit Antiserum Generation and Testing

Outbred New Zealand rabbits were injected intradermally using 250 μg of recombinant DRG1 emulsified with an equal volume of Freund's complete adjuvant. Two 125 μg intramuscular boosts of the same material in incomplete adjuvant were given 4 and 7 weeks later. Sera were collected 7 and 10 days after the last injection and tested using ELISA assays.

### Western Blotting

NIH 3T3 cells (2 × 10^6^) were lysed in 100 μl of buffer containing 0.5% NP-40, 0.1% SDS, 0.5% Na deoxycholate, 1× PBS, and protease inhibitor mix (Roche) for 15 min at 4°C, and cellular debris was removed by centrifugation. Protein (25 μg) was separated on SDS-PAGE and transferred to a nitrocellulose membrane (Bio-Rad). The membrane was blocked for 1 hr in Tris-buffered saline (25 mM Tris) plus 0.1% Tween with 5% nonfat dry milk, followed by incubation with primary antibody for 1 hr and secondary antibody for 40 min. Western blots were developed using the ECL system (Amersham, Aylesbury, UK). The polyclonal anti-MPSK1 and anti-DRG antisera were used at 1:2000 and 1:4000 dilutions, respectively. Proteins were immunoprecipitated as described (Ligos et al., 2002).

### In Vitro Protein Kinase Assay

In vitro kinase assays using purified proteins were performed as follows: 35 μl of reaction buffer containing the indicated amount of recombinant histidine-tagged MPSK1, 50 mM Tris-HCl (pH 7.4), 10 mM MnCl_2_, 10 mM MgCl_2_, 10 mM ATP, and 10 μCi/μl of [γ-^32^P]ATP, 3000 Ci/mmol (Amersham) was preincubated (30°C, 1 min) and then mixed with 10 μl of the same buffer containing the indicated amounts of the purified putative substrates. Reactions were incubated (30°C, 30 min) and then terminated by addition of SDS loading buffer, and proteins were separated using a 10% SDS-PAGE gels. After Coomassie blue staining, the gel was dried and autoradiographed.

### Determination of Peptide Phosphorylation Specificity

The consensus phosphorylation motif for MPSK1 was determined using a positional scanning peptide library approach essentially as described (Hutti et al., 2004). Reactions were carried out in multiwell plates in 50 mM HEPES (pH 7.4), 10 mM MnCl_2_, 1 mM DTT, 0.1% Tween 20, 100 μM ATP (including 0.3 μCi/μl of [γ-^33^P]ATP), 50 μM peptide substrate, and 50 μg/ml MPSK1 for 2–4 hr at 30°C. Peptides had the general sequence YAXXXXX-S/T-XXXXAGKK(biotin), where S/T represents an even mixture of serine and threonine, K(biotin) is ɛ-(biotinamidocaproyl)lysine, and X is a roughly equimolar mixture of the 17 amino acids excluding cysteine, serine, and threonine. Each well contained a distinct peptide in which one of the X positions was replaced with 1 of 20 residues (one of the unmodified amino acids excluding Ser and Thr). Three additional wells were included that contained either no peptide, the peptide YAXXXXX-S-XXXXAGKK(biotin), or the peptide YAXXXXX-T-XXXXAGKK(biotin), to examine the preference for a particular phosphoacceptor residue. At the end of the incubation time, aliquots of each reaction were spotted onto streptavidin membrane, which was washed as described and exposed to a phosphor screen (Hutti et al., 2004). Data shown are representative of four separate runs.

### Secondary Structure Prediction

The secondary structure of the kinase domain in each of the 518 human protein kinases described by Manning et al. (2002) was predicted using the following methods: Predator (Frishman and Argos, 1996, Frishman and Argos, 1997), SSPro 4.0 (Pollastri and Baldi, 2002), and PsiPred (Bryson et al., 2005). The individual predictions were combined into a consensus prediction using a majority rule using three classes: helix, sheet, and coil. In cases of a tie, the coil class was assigned. Two hidden Markov models (HMM) were built using HMMer (Eddy, 1998) using the structure-based multiple sequence alignment of 24 human protein kinases in the active state produced by Nolen et al. (2004) comprising the β6 to αF section of the kinase domain. These HMMs were used to detect the exact fragment containing the activation loop residues in each kinase sequence.

### Autophosphorylation

Reactions were initiated at 30°C by addition of 5 μM (final concentration) nonphosphorylated MPSK1 to a solution containing 50 mM HEPES (pH 7.5), 100 mM NaCl, 5 mM DTT, 5 mM ATP, and 5 mM MgCl_2_. At each time point, a 5 μl aliquot was removed and added to a buffer that terminated the reaction containing 20 mM EDTA and 0.1% formic acid. Masses of the intact protein were determined using an Agilent orthogonal time-of-flight (LC/MSD TOF) spectrometer.

### Phosphorylation Site Mapping

The protein was denatured by boiling, cysteine residues were reductively alkylated, and then a tryptic digest was performed overnight (100:1 protein:enzyme). The peptides were separated on a Dionex 3000 nano-LC system with a C_18_ Pepmap column using a water/acetonitrile gradient with 0.1% formic acid and analyzed using a Bruker HCT Ultra ion trap in MS/MS mode. Alternating fragmentation cycles were performed in data-dependent MS/MS by using collision-induced dissociation and electron transfer dissociation, the data were submitted to Mascot searches (http://www.matrixscience.com/) separately, and the information from the two alternative approaches was combined.

## Accession Numbers

The structure of MPSK1 (STK16) has been deposited in the Protein Data Bank under ID code 2BUJ.

## Supplemental Data

Document S1. Six Figures
