# Supplementary material for: Structure of the Human Protein Kinase MPSK1 Reveals an Atypical Activation Loop Architecture
Source: Structure. 2008 Jan 8;16(1):115–24. doi: 10.1016/j.str.2007.10.026 (PMC2194165; doi:10.1016/j.str.2007.10.026)
Supplement: Document S1. Six Figures [file mmc1.pdf]

# Supplemental Data

# Structure of the Human Protein Kinase MPSK1 Reveals an Atypical Activation Loop Architecture

**Jeyanthy Eswaran, Antonio Bernad, Jose M. Ligos, Barbara Guinea, Judit É. Debreczeni, Frank Sobott, Sirlester A. Parker, Rafael Najmanovich, Benjamin E. Turk, and Stefan Knapp**

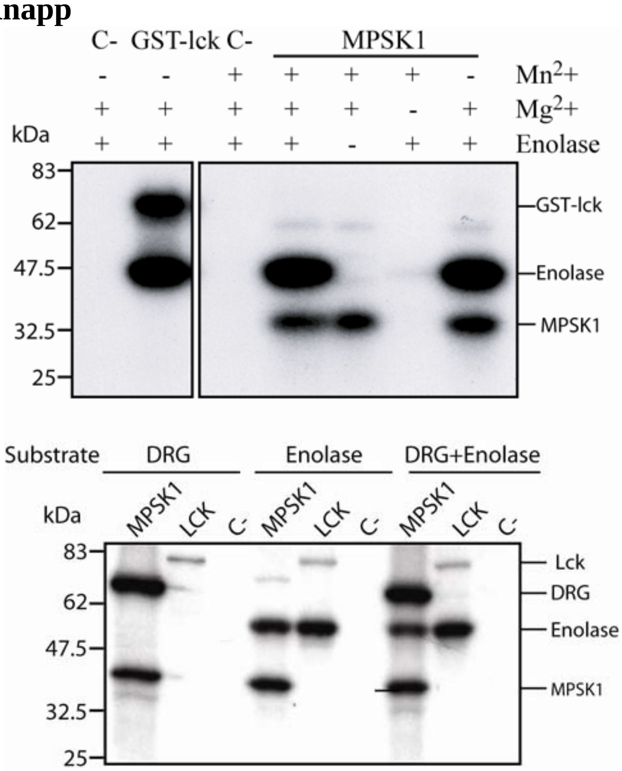


**Figure S1. Identification of MPSK1 Substrates**

Samples were separated using SDS-PAGE after *in vitro* phosporylation using recombinant MPSK1 or LCK. Incorporation of radioactive phosphate was measured using autoradiograms. LCK was arbitrarily selected to evaluate specificity for the substrates. Experiments were carried out in the presence of $Mg^{2+}$ or $Mn^{2+}$ as metal cofactors as indicated in the figure. Bacterial extracts harbouring an empty expression vector were used as negative control (C-). Panel A revealed the strong dependence of MPSK1activity on the presence of $Mg^{2+}$ for both autophosphorylation as well as phosphorylation of substrates. Panel B shows that MPSK1 but not LCK recognizes

DRG1 as a substrate whereas enolase is recognized by both kinases used. In addition, autophosphorylation of MPSK1 is evident.

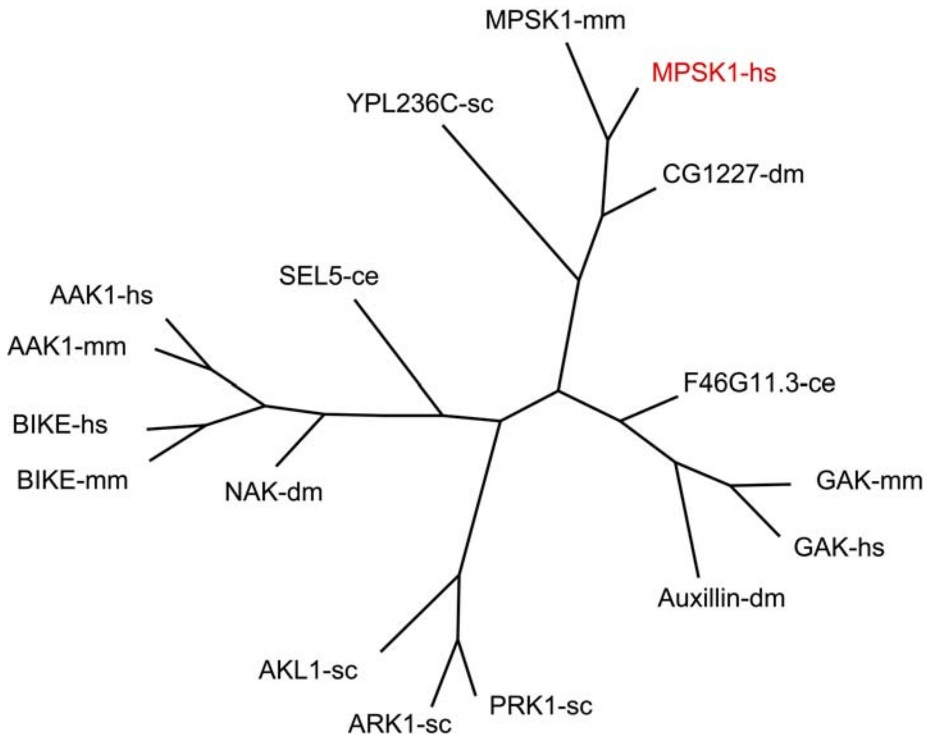


**Figure S2. Phylogenetic Tree of the NAK Family**

Sequences of NAK family members in human (hs), mouse (mm), drosophila (dm), nematode (ce) and yeast (sc) were retrieved from http://kinase.com. Sequences of the kinase domains were aligned using vector NTI.

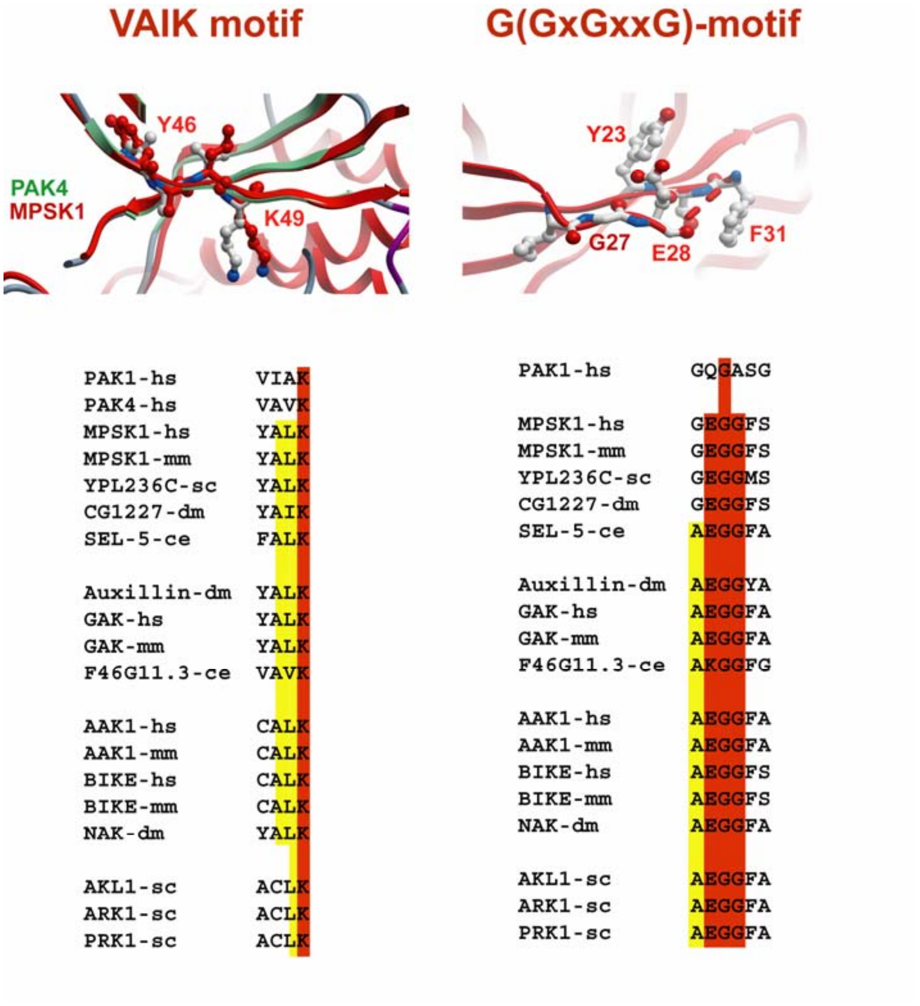


**Figure S3. Structural Conservation of the VAIK-Motif (Left Panel) and Glycine Rich Loop (Right Panel)**

The active site lysine (K49 in MPSK1) is highly conserved in the NAK family but hydrophobic residues are diverse and vary from aromatic substitutions (Tyr/Phe in MPSK1 and cysteine in AAK1 and BIKE homologues). The side chain conformations of the hydrophobic residues are however conserved and function to lock this beta sheet into position. Shown here is a superimposition of MPSK1 with PAK4 (VIAK) (pdb entry: 2J0I) as well as PAK1 (glycine rich loop) (pdb entry: 1YHV). The protein sequences are obtained from http://kinase.com and the alignment was generated using Vector NTI.

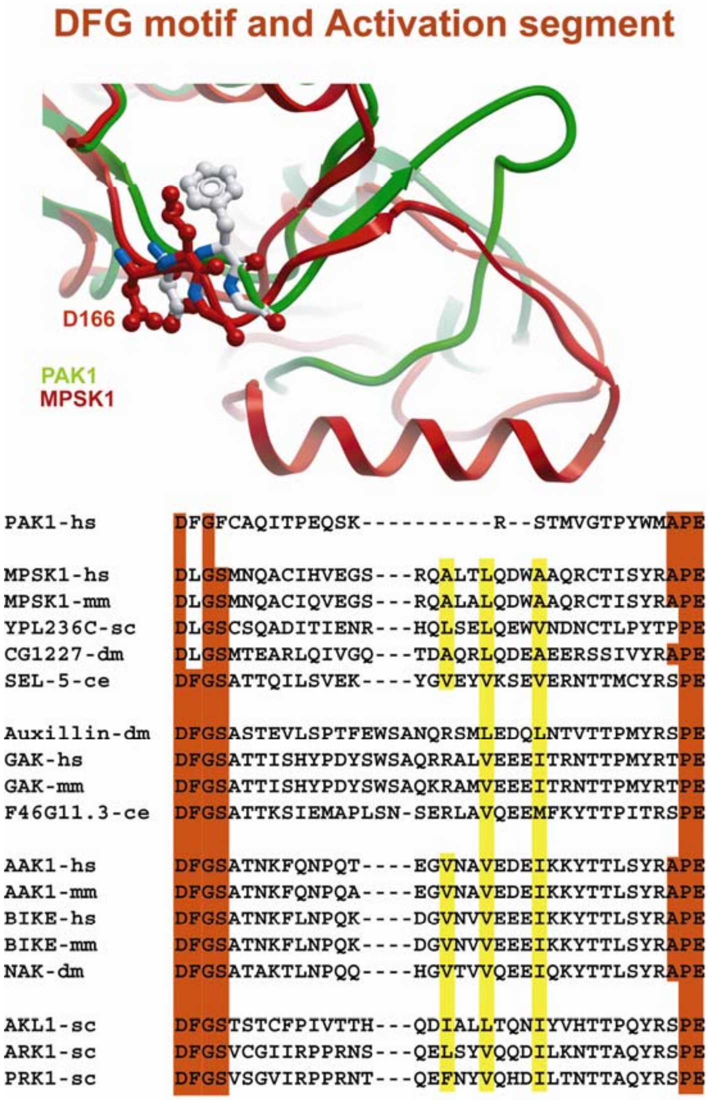


**Figure S4. Conservation of the DFG Motif and Activation Segment Residues in the NAK Family**

The DFG motif has only been altered to DLG in MPSK1 homologues. Shown here is a superimposition of MPSK1 with PAK1 (pbd entry: 1YHV). In the MPSK1 structure the lysine in DFG motif adopts the same conformation as F in PAK1. The APE motif is conserved in mammalian MPSK1 homologues as well as in AAK and BIKE. Hydrophobic residues that lead to stabilization of the activation segment helix are largely conserved except for the first residue which is absent in GAK homologues which also have a three residue insertion in this region. The protein sequences are obtained from http://kinase.com and the alignment was generated using Vector NTI.

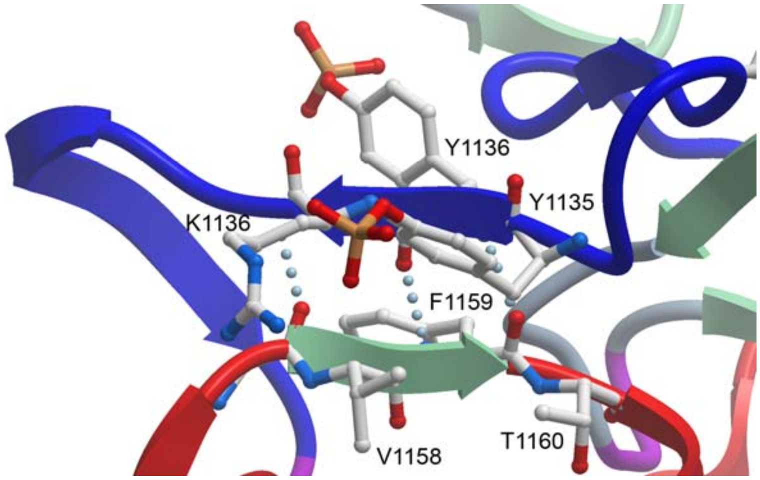


**Figure S5. Presence of a Short Antiparallel β-Sheet in Active Receptor Tyrosine Kinases**

The figure shows interaction of the antiparallel β-sheet in the activation loop of activated IGF1R receptor tyrosine kinase (pdb-entry: 1K3A). Hydrogen bonds are shown as dotted lines. Similar interactions have also been observed in other activated receptor tyrosine kinases.

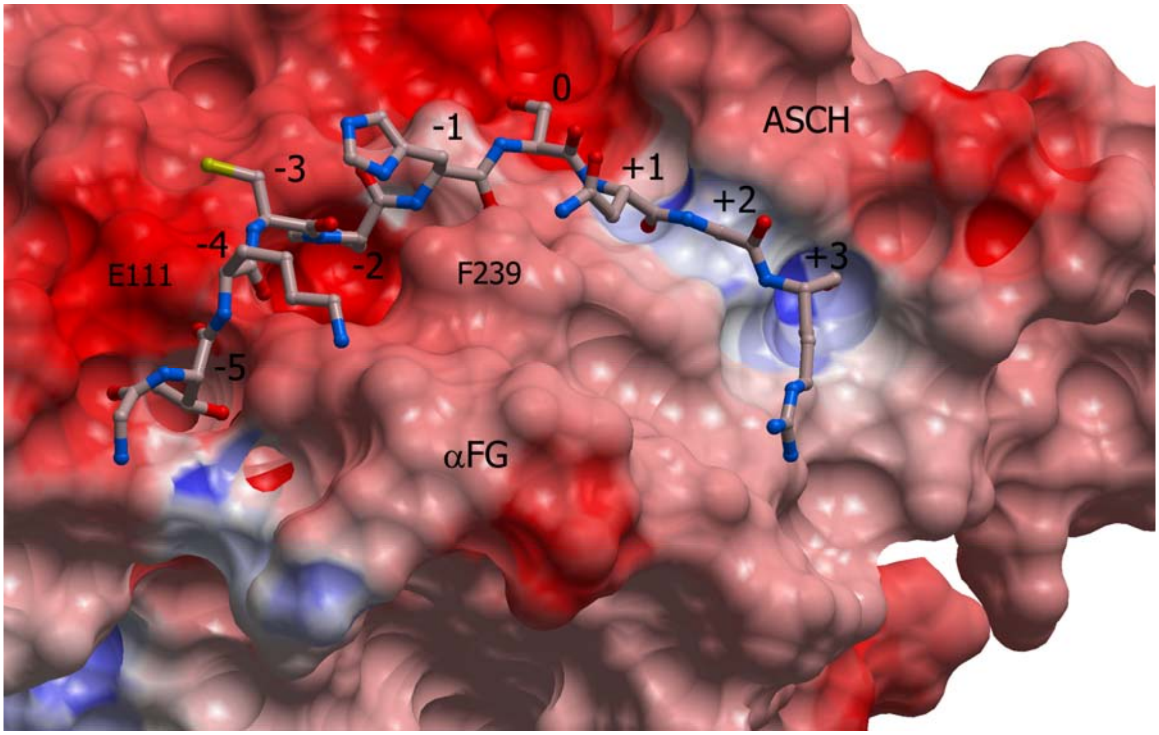


**Figure S6. Model of a Consensus Peptide Binding to the MPSK1 Peptide Binding Site**

The consensus peptide has been manually docked into the MPSK1 active site using the Co-crystal structure of PKB/Akt as a starting model[1]. The positions of substrate residue as well as MPSK1 structural elements are labelled. The surface of MPSK1 is coloured according to its electrostatic potential indicating areas of negative charge in red and positive charge in blue. The locations of the main structural elements are indicated.

[1]Yang, J., Cron, P., Good, V. M., Thompson, V., Hemmings, B. A. & Barford, D. (2002). *Nat Struct Biol* 9, 940-4.
